# Drug-resistant TB in Morobe Province, Papua New Guinea, 2012–2021

**DOI:** 10.5588/pha.24.0011

**Published:** 2024-12-01

**Authors:** L. Bumbu, S. Vaccher, A. Holmes, K. Sodeng, S.M. Graham, Y.D. Lin

**Affiliations:** ^1^ Angau Memorial Provincial Hospital, Morobe Provincial Health Authority, Papua New Guinea;; ^2^ The Burnet Institute, Melbourne, VIC, Australia;; ^3^ University of Melbourne, Department of Paediatrics, Royal Children’s Hospital, Melbourne, VIC, Australia.

**Keywords:** loss to follow-up, rifampicin-resistant tuberculosis, pulmonary, Xpert

## Abstract

**SETTING:**

Papua New Guinea (PNG) is a high-burden country for multidrug-resistant or rifampicin-resistant TB (MDR/RR-TB). There are limited data on MDR/RR-TB notifications and treatment from the most populous province.

**OBJECTIVE:**

Describe MDR/RR-TB detection and treatment outcomes in Morobe Province, the impact of the COVID-19 pandemic and factors associated with unfavourable treatment outcomes.

**DESIGN:**

Retrospective cohort study of MDR/RR-TB notifications between 2012 and 2021 using routine programme registration data. Favourable outcomes were compared to unfavourable outcomes using multivariable regression.

**RESULTS:**

Between 2012 and 2021, 160 cases of MDR/RR-TB were bacteriologically confirmed. Few diagnoses were made among children (2.5%), extrapulmonary cases (0.6%) or rural residents (38%). Case notifications rose sharply from 2016 after the introduction of GeneXpert to 5.6 cases per 100,000 population in 2020 before a reduction in 2021 coinciding with COVID-19 disruptions. Loss to follow-up (27.5%) and death (8.1%) were common. Unfavourable treatment outcomes were more common among male participants (aOR 3.00, 95% CI 1.38–6.45) and those treated with longer injectable-containing regimens (aOR 3.39, 95% CI 1.30–8.80).

**CONCLUSION:**

MDR/RR-TB detection has increased overall, but enhanced and decentralised diagnostic capacity is needed, including in important sub-populations. Persisting low treatment success rates must be urgently addressed to minimise the further emergence of drug-resistant TB in Morobe Province.

## Abstract (fr)

**CONTEXTE:**

La Papouasie Nouvelle Guinée (PNG) est un pays fortement touché par la TB multirésistante ou résistante à la rifampicine (MDR/RR-TB). Les données concernant les cas signalés et le traitement de la MDR-TB dans la province la plus densément peuplée sont insuffisantes.

**OBJECTIF:**

Présenter les résultats liés à la détection et au traitement de la MDR/RR-TB dans la province de Morobe, en tenant compte de l'impact de la pandémie de COVID-19 ainsi que des facteurs contribuant aux résultats de traitement défavorables.

**MÉTHODES:**

Étude de cohorte rétrospective des notifications de MDR-TB signalés entre 2012 et 2021, basée sur les données des programmes de surveillance habituels. Les résultats positifs ont été mis en relation avec les résultats négatifs à travers une régression multivariée.

**RÉSULTATS:**

Entre 2012 et 2021, un total de 160 cas de MDR/RR-TB ont été confirmés bactériologiquement. Les diagnostics chez les enfants étaient rares, représentant seulement 2,5% des cas, tandis que les cas extrapulmonaires et ceux chez les résidents des zones rurales étaient respectivement de 0,6% et 38%. À partir de 2016, une augmentation significative des notifications de cas a été observée, suite à l'introduction du système GeneXpert, atteignant 5,6 cas pour 100 000 habitants en 2020, avant de diminuer en 2021, en raison des perturbations causées par la pandémie de COVID-19. Les pertes de suivi étaient fréquentes, atteignant 27,5%, tout comme les décès, qui représentaient 8,1%. Les résultats du traitement étaient moins favorables chez les hommes (aOR 3,00 ; IC à 95% 1,38–6,45) et chez ceux recevant des traitements prolongés incluant des injectables (aOR 3,39 ; IC à 95% 1,30–8,80).

**CONCLUSION:**

La détection de la MDR/RR-TB a globalement progressé, cependant, il est impératif de renforcer et de décentraliser les capacités de diagnostic, notamment au sein de sous-populations significatives. La persistance de faibles taux de réussite des traitements doit être traitée de toute urgence afin de réduire l'émergence de la TB résistante aux médicaments dans la province de Morobe.

TB is a critical global health problem, with an estimated 10.6 million people developing TB in 2022.^1^ In 2022, 175,650 people were diagnosed and treated for multidrug-resistant/rifampicin-resistant TB (MDR/RR-TB) globally, but low detection and treatment coverage remains a major challenge as this is equivalent to only about 40% of those estimated globally to have MDR/RR-TB. Papua New Guinea (PNG) is one of the top 30 high-burden countries globally for TB and MDR/RR-TB, with case notification rates of respectively 432 cases and 22 cases/100,000 in 2022.^1^ MDR/RR-TB is a growing public health issue in PNG accounting for 3.6% of new and 22% of previously treated TB cases in 2022.^1^ The treatment success rate for 229 people who commenced MDR/RR-TB treatment in 2021 was 72%.^1^ However, there are limited MDR/RR-TB data from PNG outside the recognised ‘hot spot’ districts of the National Capital District and the South Fly District of Western Province.^2–4^

Morobe is the largest province in PNG, with an approximate population of over 1.1 million. In 2016, the TB case notification rate for Morobe Province was 529/100,000.^5^ Access to MDR/RR-TB diagnosis has improved in PNG since the implementation of GeneXpert (Cepheid, Sunnyvale, CA, USA) for diagnosis in 2012,^6^ but the proportion of bacteriologically confirmed TB remains low (28%).^7^ In a cross-sectional study performed in 2012–2014, the percentage of people diagnosed with MDR/RR-TB was 13.3% in a large urban hospital compared to 3.1% in rural Morobe.^3^ The WHO-recommended MDR/RR-TB treatment regimens have changed over the last decade, with a transition from long regimens of 18 months or more to shorter (9–12 months) regimens.^8,9^ In Morobe Province, treatment of MDR/RR-TB followed national TB guidelines, including a switch from a long regimen to a shorter regimen in 2017, but an injectable aminoglycoside has remained as part of the current regimens.

While cases were first detected in 2020, PNG experienced its first major wave of the COVID-19 pandemic in 2021, resulting in major disruptions to TB diagnosis and treatment, with clinic closures, re-prioritisation of staff and the use of GeneXpert instrument for detection of SARS-2-CoV.^10^ Total TB case notifications in 2020 decreased globally and in PNG by 18% and 6.7%, respectively, compared to 2019.^1,10^ In 2022, there were 175,650 MDR/RR-TB case notifications globally, which is below the pre-pandemic level of 181,533 in 2019.^1^

Given the lack of data on the epidemiology, clinical presentation and treatment outcomes of MDR/RR-TB in PNG outside Western Province and National Capital District, we aimed to evaluate MDR/RR-TB notification data in Morobe Province. The study describes changes to TB treatment between 2012 and 2021, the impact of the COVID-19 pandemic (2020–2021) on MDR/RR-TB case detection and treatment outcomes, and factors associated with unfavourable treatment outcomes.

## METHODS

### Study setting and population

Morobe Province is the most populous province in PNG, with an estimated population of 1.1 million who predominantly live outside the provincial capital, Lae.^11^ It has 53 reporting health facilities, including hospitals and small health centres, and 342 Aid posts (although less than half are functional) across nine districts. Angau Provincial Hospital is the only programmatic management of drug-resistant TB (PMDT) site that provides treatment for people with MDR/RR-TB. The first person with MDR/RR-TB was diagnosed in 2012.

Adults and children with presumptive TB have provided samples for smear microscopy and Xpert MTB/RIF (Xpert; Cepheid, Sunnyvale, CA, USA) assay since 2016. Samples sent for Xpert assay included sputum and gastric aspirate for pulmonary TB and lymph node aspirate, cerebrospinal fluid, pleural fluid or ascites for extrapulmonary TB (EPTB) diagnosis. If rifampicin resistance was detected, the sample was sent to the TB reference laboratory in Port Moresby for culture and phenotypic drug susceptibility testing (DST).

Drug regimens followed National TB Programme guidelines at the time of treatment initiation. All patients received inpatient treatment for the period the injectable was administered, up to 8 months under the longer regimen. The shorter regimen with injectable contained levofloxacin, prothionamide, clofazimine, ethambutol, pyrazinamide and isoniazid for 11 months, supplemented by an injectable (such as amikacin or kanamycin) in the intensive phase. The drugs in the longer regimen with injectable changed over time; this regimen currently consists of amikacin with five or six other drugs.

This study population was people of all ages diagnosed with MDR/RR-TB who were started on treatment in Morobe Province between January 2012 and December 2021.

### Study design

This is a retrospective descriptive cohort study of MDR/RR-TB notifications in Morobe Province in PNG between 2012 and 2021.

### Data collection and analysis

Data was collected from the PMDT and laboratory registers. The data variables collected included sex, age, urban/rural residence, HIV status, registration category, site of TB disease, TB resistance type, regimen used for treatment and treatment outcomes. The standard definitions of treatment outcomes were used as defined in the national and international TB guidelines.^13^

Data were extracted and analysed using Stata v17 (StataCorp, College Station, TX, USA). Categorical variables, such as sex, type of TB, and location, are presented using frequencies and percentages. Continuous variables, like age, are reported as medians with interquartile ranges (IQRs). The MDR/RR-TB notification rate has been calculated using population estimates for Morobe Province for each year.

Treatment outcomes were categorised as favourable (completed or cured) or unfavourable (loss to follow-up [LTFU], treatment failure or death). Patients with outcomes ‘not evaluated’, including those transferred for care outside Morobe Province, were excluded from the regression analysis. Associations between unfavourable treatment outcomes and variables were assessed using univariate and multivariate analyses. We employed an odds ratio (OR) and a 95% confidence interval (95% CI), and statistical significance was determined by the *P*-value being less than 0.05.

### Ethics approval

Ethics approval was obtained from the PNG Medical Research Advisory Council, Port Moresby, PNG, for this study.

## RESULTS

**FIGURE 1.**
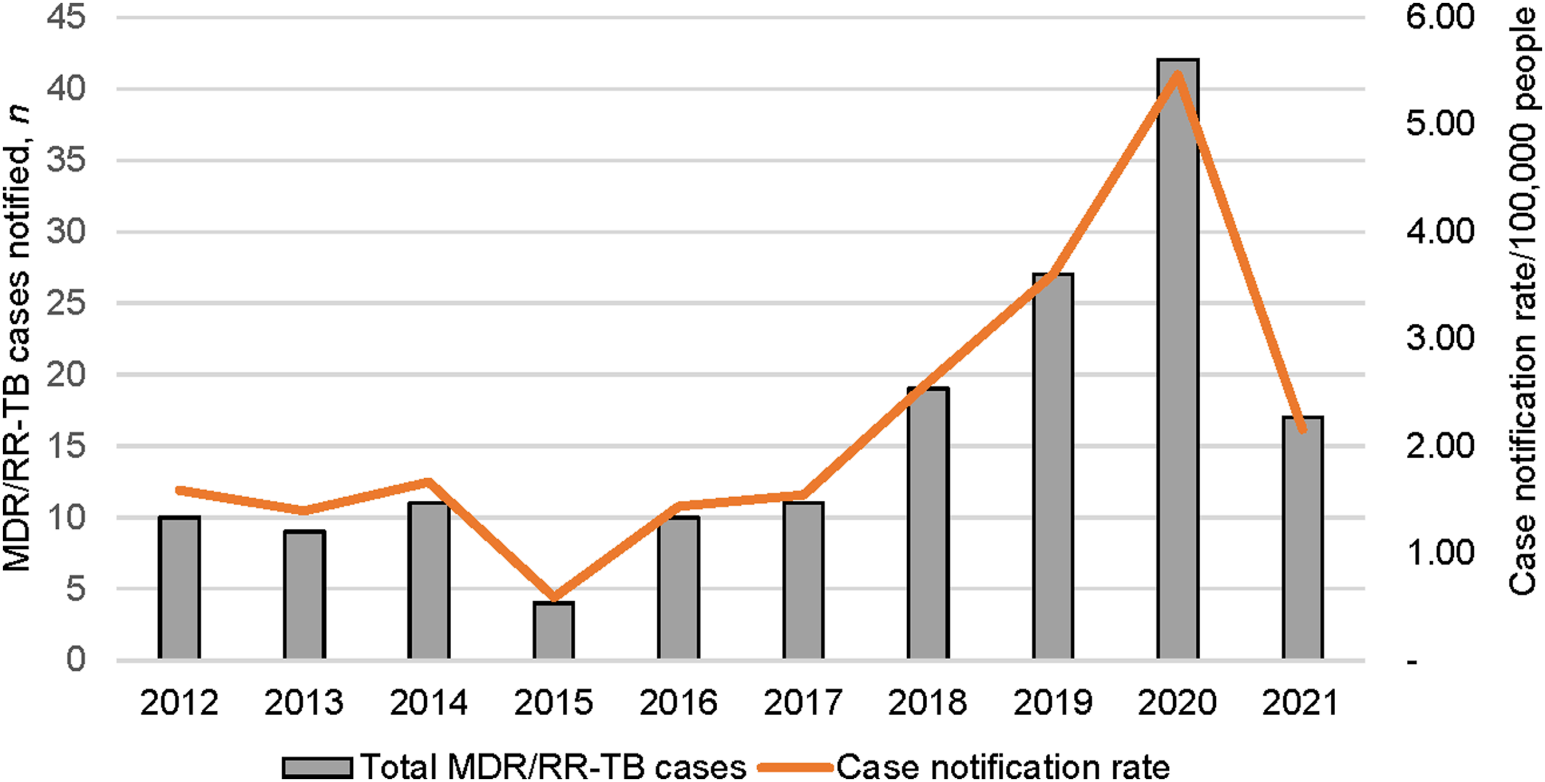
Total MDR/RR-TB notifications and case notification rates, Morobe Province, PNG, 2012–2021. MDR/RR-TB = multidrug-resistant or rifampicin-resistant TB; PNG = Papua New Guinea.

Between 2012 and 2021, 160 people were diagnosed with MDR/RR-TB in Morobe Province. The median age was 33 years (IQR 24–40). The trend of MDR/RR-TB cases as per the PMDT register was stable between 2012 and 2015, then increased year-on-year between 2016 and 2020 to peak at 42 cases (case detection rate of 5.6/100,000, 1% of total TB cases) before a substantial decline in MDR/RR-TB cases registered in 2021 (Figure 1).

**FIGURE 2.**
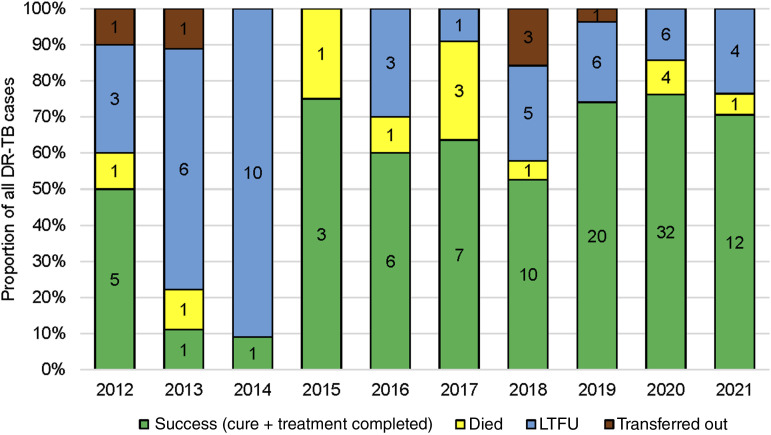
Treatment outcomes for MDR/RR-TB cases by year of treatment commenced, Morobe Province, PNG, 2012–2021. Note: outcome dates are based on the treatment-starting cohort year, not the date the outcome was reported. The numbers shown on the bars are the number of participants in each category. DR-TB = drug-resistant TB; LFTU = loss to follow-up.

All MDR/RR-TB cases were bacteriologically confirmed as rifampicin-resistant, primarily using Xpert MTB/RR rather than phenotypic DST following culture. More than half of the cases were male, and 29% were aged between 25 and 34 years (Table 1). The majority of people with MDR/RR-TB resided in urban areas. Only 2.5% were children (<15 years), and only one case of EPTB was diagnosed with TB lymphadenitis.

**TABLE 1.** Characteristics of MDR/RR-TB cases notified in Morobe Province, PNG, 2012–2021.

Characteristics	Total *n* (%)
Total, *n*	160
Age, years	
Median [IQR]	33 [24–40]
0–14	4 (2.5)
15–24	38 (23.8)
25–34	47 (29.4)
35–44	37 (23.1)
≥45	34 (23.1)
Sex	
Female	70 (43.8)
Male	90 (56.2)
Residence	
Lae	98 (61.3)
Outside Lae but inside Morobe	61 (38.1)
Unknown	1 (0.6)
HIV status	
Negative	86 (53.8)
Positive	7 (4.4)
Unknown	67 (41.9)
TB type	
EPTB	1 (0.6)
PTB	159 (99.4)
Treatment regime	
Long regimen (18–24 months with injectable)	62 (38.8)
Short regimen (9–11 months with injectable)	98 (61.3)
Type of drug resistance	
MDR-TB using DST/culture	12 (7.5)
RR-TB on GeneXpert	148 (92.5)
Treatment outcomes	
Lost to follow-up	44 (27.5)
Cured	3 (1.9)
Death	13 (8.1)
Transfer	6 (3.8)
Treatment completed	94 (58.8)
Registration	
New	98 (61.3)
Previously treated	62 (38.8)

MDR/RR-TB = multidrug-resistant or rifampicin-resistant TB; PNG = Papua New Guinea; EPTB = extrapulmonary TB; PTB = pulmonary TB; DST = drug susceptibility testing; IQR = interquartile range.

While only a low proportion of MDR/RR-TB was diagnosed in people living with HIV (4%), HIV status was unknown for 42%. Before 2019, most people diagnosed with MDR/RR-TB in Morobe were previously treated cases; from 2019 onwards, new cases predominated. When comparing people with unfavourable treatment outcomes to those who were cured or completed treatment, males were significantly more likely than females to have an unfavourable treatment outcome (Table 2). From 2018 onwards, most people were treated with a shorter regimen of 9–11 months. Over the 10-year period, unfavourable treatment outcomes decreased from 91% in 2014 to 22% in 2019. However, there was a slight increase in 2020 and 2021 to 24% and 29%. Individuals on the previous longer (18–24 months) injectable-containing regimen were less likely to have a favourable treatment outcome compared to those on the newer, shorter (9–11 months) regimen (45.8% vs 73.7%; OR 0.3, 95% CI 0.15–0.6). Risk factors of male sex and longer regimen with injectable remained statistically significant in the multivariate analysis.

**TABLE 2.** Factors associated with unfavourable outcomes among people treated for MDR/RR-TB in Morobe Province, PNG, 2012–2021.*

	Unfavourable (*n* = 57) *n* (%)		Favourable (*n* = 97) *n* (%)		Total (*n* = 154)* *n* (%)		OR (95% CI)	*P* value	aOR (95% CI)	*P* value
Age category, years										
<25	10	17.5	31	32.0	41	26.6	Ref			
25–44	33	57.9	48	49.5	81	52.6	2.13 (0.92–4.93)	0.08	1.67 (0.66–4.22)	0.28
>45	14	24.6	18	18.6	32	20.8	2.41 (0.89–6.54)	0.08	2.23 (0.74–6.74)	0.16
Sex										
Female	16	28.1	50	51.5	66	42.9	Ref			
Male	41	71.9	47	48.5	88	57.1	2.73 (1.35–5.50)	0.01	3.00 (1.38–6.45)	0.01
Residence										
Lae	30	52.6	64	66.0	94	61.0	Ref			
Morobe	26	45.6	33	34.0	59	38.3	1.68 (0.86–3.29)	0.13	1.91 (0.89–4.10)	0.1
Unknown	1	1.8	0	0.0	1	0.6				
HIV status										
Negative	36	63.2	46	47.4	82	53.2	Ref			
Positive	2	3.5	5	5.2	7	4.5	0.51 (0.09–2.79)	0.43	0.71 (0.12–4.72)	0.72
Unknown	19	33.3	46	47.4	65	42.2	0.53 (0.26–1.05)	0.07	0.67 (0.26–1.73)	0.41
Regimen										
Long	32	56.1	27	27.8	59	38.3	Ref			
Short	25	43.9	70	72.2	95	61.7	0.30 (0.15–0.60)	0.001	0.30 (0.11–0.77)	0.01
Type of resistance										
MDR-TB	6	10.5	6	6.2	12	7.8	Ref			
RR-TB	51	89.5	91	93.8	142	92.2	0.56 (0.17–1.83)	0.34	1.25 (0.32–4.84)	0.75
Registration										
New	31	54.4	66	68.0	97	63.0	Ref			
Previously treated	26	45.6	31	32.0	57	37.0	1.79 (0.91–3.5)	0.09	0.72 (0.28–1.85)	0.5

* Six patients with outcome of ‘transferred’ were excluded from the analysis as they were ‘not evaluated’.

MDR/RR-TB = multidrug-resistant or rifampicin-resistant TB; PNG = Papua New Guinea; OR = odds ratio; CI = confidence interval; aOR = adjusted OR; Ref = reference.

## DISCUSSION

The study findings suggest major challenges for case detection or treatment coverage of MDR/RR-TB in Morobe Province. Overall, case notifications were low until 2016, after which there was a sharp increase following the introduction of GeneXpert. The fall in 2021 may reflect COVID disruptions, so monitoring case detection trends in 2022 will be necessary. Higher numbers from the urban setting may partly reflect limited access to diagnostics in rural areas. All MDR/RR-TB were bacteriologically confirmed, but there is likely an underestimate of the burden, as highlighted in our study by the very low numbers of children or extrapulmonary cases, where there is increased difficulty attaining bacteriological confirmation. Although treatment success has improved as the caseload has increased, treatment success rates are well below international targets. The study findings are concerning, especially given that the epidemiology of MDR/RR TB in PNG is not well known outside the National Capital District and Western Province. Morobe province is PNG’s largest province and, from Lae, serves as a gateway to the Highlands, Wau and Madang (with combined populations of 3.5 million people) via the Highlands Highway.^12^

The male predominance with most cases in young people (15–45 years old) is consistent with TB epidemiology in PNG.^5^ Children (<15 years) represent 35% of the Morobe population but represent only 2.5% of MDR/RR-TB diagnoses. Underdiagnosis in children is common in many settings,^1^ especially as bacteriological confirmation often requires performing gastric aspirates and non-respiratory samples for children with MDR/RR-TB, especially young children (<5 years). Community based active contact investigation of households of people with bacteriologically confirmed MDR/RR-TB is required for detection of new cases and provision TPT for eligible contacts.^14,15^ Screening for people with presumptive MDR/RR-TB, greater access to rapid molecular diagnostics, including in rural settings, training of healthcare workers in clinical skills required for bacteriological diagnosis and introduction of contact investigation are important steps to close major gaps in detection and improve treatment outcomes.

Although living in urban settlements is a known risk factor for MDR/RR-TB, the finding that the majority of people diagnosed with MDR/RR-TB resided in urban areas of Morobe may suggest that there may be higher primary transmission or secondary acquisition of MDR/RR-TB in the urban areas, which needs further evaluation. Alternatively, it may indicate under-detection of MDR/RR-TB in rural areas due to geographic challenges such as limited access to health facilities, limited GeneXpert sites, and suboptimal specimen referral networks. Sputum selection bias occurs as only well-resourced centres send specimens to the reference labs.^2^ However, it may also be an overestimate as people travelling from rural areas to central facilities may report their most recent residence rather than their usual one.

The proportion of MDR/RR-TB cases notified compared to total TB cases (1% in 2020) is possibly underestimated as less than half of overall TB cases are bacteriologically confirmed in Morobe. This could be due to sputum not being sent for Xpert testing^3^ or challenges in obtaining specimens for bacteriological confirmation, especially in children, as noted. Similarly, while EPTB makes up almost half the cases of drug-susceptible TB,^5^ there is probably an under-detection of drug-resistant EPTB cases. Bacteriological confirmation requires procedural skills (such as performing fine needle aspirate, gastric aspirate or lumbar puncture) and equipment different cadres, such as staff at rural posts, may not have. On the other hand, PNG could consider utilising novel diagnostic techniques such as a stool sample for GeneXpert testing for child diagnosis.

Registration and data since 2018 show more cases of newly diagnosed MDR/RR-TB as opposed to retreatment cases. This may be due to ascertainment bias, as from 2012 to 2016, only people with risk factors for MDR/RR-TB (TB treatment failures, contacts of MDR-TB patients and people living with HIV were tested using Xpert.^2^ From 2017, Xpert was recommended as a frontline test for all people with presumptive TB. However, the predominance of people diagnosed with MDR/RR-TB who are new cases suggests there is primary community transmission, as previously mentioned. Rarely were any DST results received due to logistical constraints; because of limited access to DST/culture, there is no reliable way to detect fluoroquinolone resistance or pre-XDR-TB (pre-extensively drug-resistant TB), making both regimens insufficient.

This study found an increase in treatment success since 2015. This may be partially explained by the introduction of a shorter regimen that has been required for patients since 2017. The Standard Treatment Regimen of Anti-Tuberculosis Drugs for Patients with MDR-TB [STREAM] clinical trial showed lower LTFU in those on the shorter regimen than in those on longer regimens.^16^ A recently published qualitative study with people who were LTFU to MDR/RR-TB treatment showed the duration of treatment and use of injectables to the most challenging aspects of treatment.^17^ Unfavourable outcomes reported in this study were particularly high from 2013 to 2014 (90%), with high rates of LTFU (80–90%). This compares with the treatment outcomes data from Daru, Western Province, in 2014, where unfavourable outcomes of 30% were reported, including 18% being LTFU.^17^

This study found that the male sex was a significant risk factor for unfavourable treatment outcomes. This has been reported in other settings.^18,19^ Other studies have shown a correlation between unfavourable treatment outcomes and age; however, age was not a significant risk factor in this study. Shorter regimens appeared to be correlated with less unfavourable treatment outcomes, probably driven by less loss to follow-up. We were not able to assess how long people received inpatient care, which is relevant to understanding LTFU. Financial challenges may be associated with prolonged admission in a country where >80% of people rely on subsistence farming. Global studies show that factors such as income, lack of social support and as well as self-perception at low or no risk for TB relapse are associated with LTFU.^20,21^ A study from Madang with drug-susceptible TB (DS-TB) patients found that high LTFU rates were linked to travel time of >3 hours to the health facility.^22^ A study in Daru shows that addressing psychosocial risk factors with education and counselling could improve treatment retention.^23^ The ability to receive decentralised treatment, similar to DS-TB, may reduce LTFU.

A study limitation is that the rate of pre-treatment LTFU was not determined due to a lack of access to paper laboratory registers. WHO has recently updated the definition of LTFU to include those diagnosed with TB who never commenced treatment.^13^ Programmatic reporting of pre-treatment LTFU is crucial in analysing gaps in the TB diagnostic cascade and linkage to care. Another important limitation is that lack of access to diagnostics and current diagnostic practices do not allow us to determine the true burden of MDR/RR-TB in Morobe Province, especially among some key vulnerable populations. Furthermore, this retrospective study was unable to identify independent, potentially modifiable factors that impact case detection and may have informed policy and practice changes.

This study did not examine adverse effects by regimen; however, severe adverse effects of longer and shorter regimens with injectables were common in the STREAM 1 trial (45.4% versus 48.2%, respectively).^16^ A study in PNG showed that 27% of those treated had toxicity using the short injectable regimen.^24^ However, the reclassification of medication categories over time to include more effective drugs (even in the longer regimen) probably contributed to improved treatment outcomes. For example, repurposed drugs such as linezolid and clofazimine were introduced in 2016, and kanamycin was replaced by amikacin, the only aminoglycoside with efficacy. The WHO recommends new 6-month all-oral regimens containing bedaquiline and linezolid (BPaL/M) with higher treatment success rates (89% in TB PRACTECAL).^25^ The implementation of oral drugs with the potential of decentralisation will contribute to less LTFU and better treatment outcomes.

In conclusion, our study findings highlight the need for active case finding and strengthening diagnostic capabilities in Morobe Province to detect and treat people with MDR/RR-TB. This includes decentralisation of TB services to primary or rural levels of care as well as improving the diagnosis of MDR/RR-TB in children or people with EPTB. High rates of unfavourable outcomes, especially LTFU, are a major concern. Our findings support the introduction of shorter, all-oral treatment regimens for MDR/RR-TB treatment that are well tolerated, as well as measures to reduce the catastrophic psychosocial, financial and physical costs associated with treatment.
